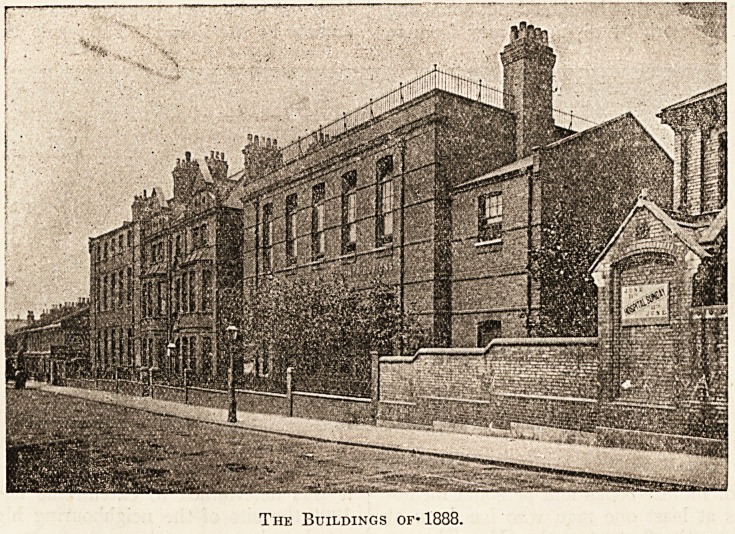# The Growth of the West Ham Hospital

**Published:** 1911-07-08

**Authors:** 


### HOSPITALS OF TO-DAY.

### IX.—THE GROWTH OF THE WEST HAM HOSPITAL.

The West Ham and Eastern General Hospital, with the exception of the Poplar Hospital for Accidents and one other institution, is, so an advertisement might read, the only general hospital for adults in East London. The change that has come over its work, the extension of its size and importance, was summarised, as The Hospital noted on March 18 last, in a change of name. It has outgrown its beginning as the West Ham Hospital, and the area from which its patients come is recognised by the addition of the words "Eastern General" to its title. When its whole story comes to be written, from its beginning in 1861 as a dispensary in Romford Road to its present position as a hospital with a hundred beds, no lack of historical material will be found worth preserving, and we sincerely suggest to Mr. Thomas Alexander Cook, the present Chairman, that he should put some day the crown on his work by giving literary form to his collection of minute-books and personal memories. There is at least one man who has been at the hospital from the first—namely, Mr. Thomas Lloyd, the dispenser, who has held that post since 1871, and his experience of the work was epitomised recently in the remark that in the old days he had about thirty-seven patients a day, and that now he feels himself lucky if he gets off with 350. The hospital's recent history, however, is all that must detain us here.

The foundation-stone of the buildings in Bryant Street, which with their south aspect form the front of the hospital, was laid by the Duke of Cambridge in 1888, and six years later the Passmore Edwards wing was added. These additions gave the hospital a total of sixty beds, but it is with the beginning of the new century that its growth really takes on a new phase. The year 1900 moreover coincided with the appointment of certain new members of the staff. Mr. A. W. Scrivener, the present Secretary, was chosen in this year, and in 1901 Mr. T. A. Cook, who had been on the committee for a long period, was elected Chairman of the Board of Management for the first time. The hospital's affairs at this date were somewhat critical, and the new chairman took the opportunity afforded him by proposing, and eventually carrying, certain important resolutions which placed the administration on a more up-to-date and satisfactory basis. While these internal changes were taking place the problem of accommodation was growing monthly more pressing. At present the hospital serves a population of something like a million, and up to 1905 it could make only a very inadequate return to the demands upon its beds. It is hardly necessary to remind our readers that West Ham is a district which pullulates with factories, and that the inevitable number of accidents attendant on them has been increased by the motor-omnibus traffic. The effects of air-transport on the hospital have yet to be seen, but they are awaited with some anxiety by the Board. The result was that in 1904 it was determined to extend the hospital, and in 1905 the site of the neighbouring high school was purchased—an opportune moment, as it happened, since the hospital, as the new proprietor, was able to obtain compensation from the local authorities for the effects of a broadening of the adjoining thoroughfare which was being undertaken at the time. But the difficulties in the hospital's path, after a site had been secured, had only begun. As Mr. Cook remarked, "We were told by a very eminent hospital authority that there was nothing to be done but raze the old buildings to the ground and build on a new plan. But that would have entailed leaving West Ham without a hospital bed for a considerable length of time, and the committee therefore decided to endeavour to make the best of the old building in adding on the new, and surmounted some great architectural difficulties in so doing." The results are before us. On the ground and first floors steps have been made to efface themselves into easy slopes, and a problem of altering levels consequent on this arrangement has been cleverly solved. The result has been that patients can be wheeled to the

The Recent Extension.

theatre without the jolts and risks of passing down steps on the way.

By the purchase of the site of the high school just referred to the land on which the hospital stands has been enlarged roughly from an oblong into a square. The hospital is built at a corner, along the angles of which its buildings extend, and the additions of the 1906 period can be summarised by saying that quarters for the house surgeons have been added to one extremity, while what is virtually a new pavilion, consisting of two wards, and above them sleeping-rooms for nurses, has been built across the centre of the open square at the back. The effect of this is to secure cross ventilation to every ward. The position of the old kitchen, too, was an important factor in this scheme. Placed at the rear of the original wards, the kitchen has been made the centre of the whole site, and the new pavilion is served directly from it.

The general effect of the extension thus outlined

A few words should be said concerning the resident medical officers' quarters, which form more or less a block to themselves. On the floor above them has been placed an isolation ward, which is not yet used, since the King's Fund, which recently gave £500 to the hospital, laid it down that if the two purposes were to be provided for in one building, two staircases were essential. At present there is only one, and the best way of adding a second is now under consideration by the Board. The architect is Mr. Percy Adams.

The general reconstruction which has taken place has allowed better accommodation for the nurses, and also, we believe, for the domestic staff. At present, however, it is not found possible to allow to each nurse a room to herself; and the housing of the wardmaids also requires some management.

Figures are not allowed to intrude into this series of articles further than is essential, and so the nature of the support which the hospital gains must be

The Buildings of 1888.

must now be supplemented. Besides the new pavilion, a new theatre has been added. Its construction on a position with a north light has been felt as a great improvement on its original situation, which, we understand, was said by one operator to ventilate directly on to the rubbish heap. To-day, of course, it is the regulation unit with anæsthetising and sterilising rooms. The administrative offices are also new, so is the dispensary, and the necessarily bare structure of the mortuary has been lightened by the reliefs which the Kyrle Society recently presented for its decoration. On one of the old wards has been built a new storey, which contains the sisters' rooms. Last, and in a sense chief, is the reconstructed out-patient department. This is presided over by a lady almoner, a new appointment, held by Miss Gregory since January 1 of this year. We understand that the value of such a post has proved itself once again at West Ham.

stated generally. The King's Fund has been a prominent subscriber to and critic of the hospital. Here as elsewhere annual subscriptions have been falling off, and here too the contributions of the working-men—and it is obviously a working-man's district—are gradually improving. The West Ham and Eastern General Hospital has not been favoured liberally with legacies; last year they amounted to £500. About £7,000 a year is required to maintain the hospital. The probable effect of the Insurance Bill on the hospital was discussed by Mr. Cook in our issues of June 3 and 10, pages 245 and 268 respectively.

The wants of the hospital will have been somewhat foreshadowed by what has been written above. One of the most important is a nurses' home. A site close at hand is therefore wanted. Were this home built it would probably be possible to complete the improvements already made in the domestic staff's accommodation. At present, however, we understand there is always the possibility that some of the nurses may have to sleep out. A balcony for the children's ward is also being installed and the ward re-decorated and tiled, and it is hardly to be expected that those responsible for the women's medical ward, which is complementary to it, will not then press their claim for a balcony also. The balcony is a gift from the Duchess of Marlborough, and the re-decoration from the West Ham School Children's Hospital Fund. A surgeon's laboratory, with a room for microscopic work, at some future date will probably be required, and, perhaps, a skin clinic with Röntgen apparatus; but the nurses' home and the additional staircase already mentioned for the resident medical officers' block are the principal needs at present.

In conclusion it may be added that a great deal of energy and enthusiasm have been excited among the hospital's workers, whose institution after all is not as widely known to the general public as its sister a little further west. This perhaps has been an incentive, and certainly a praiseworthy example has been set by the hospital's patron, the Duchess of Marlborough, in memory of whose generosity one of the new wards has been named, and a sample of whose interest may be gathered from the record of her visit, which was published in The Hospital last week.

## Figures and Tables

**Figure f1:**
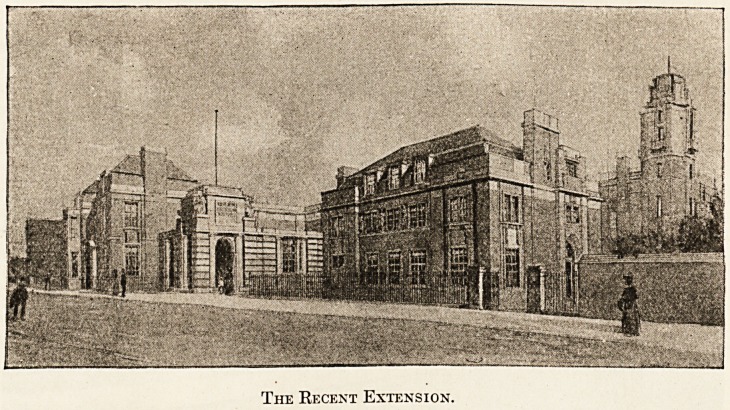


**Figure f2:**